# Therapeutic Effects of CDK4/6 Inhibitors in Gastric and Colonic Metastases From Breast Cancer: A Case Report

**DOI:** 10.7759/cureus.52765

**Published:** 2024-01-22

**Authors:** Akinori Sasaki, Shuko Masuda, Tsubasa Yoshioka, Akira Saito, Yasuaki Motomura

**Affiliations:** 1 Gastroenterology, Tokyo Bay Urayasu Ichikawa Medical Center, Urayasu, JPN; 2 Pathology, Tokyo Bay Urayasu Ichikawa Medical Center, Urayasu, JPN

**Keywords:** gastric and colorectal metastases, palbociclib, cdk4/6 inhibitor, lobular carcinoma, breast cancer

## Abstract

Breast cancer often metastasizes to the lungs, bones, liver, and brain; however, gastric and colonic metastases from breast cancer are rare. Nevertheless, here, we present the case of a 50-year-old woman diagnosed with recurrent breast cancer, exhibiting gastric and colonic metastases that were detected when she experienced intermittent abdominal pain. The differentiation between primary gastric cancer and metastasis from breast cancer was made through immunohistochemical staining. The patient underwent treatment with palbociclib, a cyclin-dependent kinase (CDK)4/6 inhibitor, and anastrozole, with no significant adverse effects. Subsequent upper and lower endoscopic examinations following the initiation of these treatments revealed tumor shrinkage in both gastric and colonic metastases. This case report presents the first instance in which morphological changes in gastrointestinal metastasis induced by CDK4/6 inhibitors could be evaluated.

## Introduction

Breast cancer is the most prevalent cancer worldwide, including in Japan [1]. In early-stage breast cancer, surgical intervention typically leads to a complete cure. However, the rate of local and distant recurrence ranges from 10% to 20% at five years, and 20% to 30% within 10 years post-treatment [2]. Distant metastases primarily manifest in sites such as the lungs, bones, liver, and brain [3], while occurrences in the gastrointestinal (GI) tract, specifically the stomach and colon, are notably uncommon. Diagnosing gastric and colonic metastases is challenging because of their rarity, which is further complicated by the extended disease-free interval between the initial breast cancer diagnosis and the eventual detection of GI metastases [4].

Patients diagnosed with hormone receptor-positive metastatic breast cancer typically undergo endocrine therapy (ET). However, recent advancements in treatment approaches have led to the adoption of combination treatments, involving the addition of a cyclin-dependent kinase 4 and 6 (CDK4/6) inhibitor to ET. This treatment combination has demonstrated remarkable therapeutic effects and has gained approval for patients with hormone receptor-positive metastatic breast cancer [5]. Notably, a higher cytoreductive effect has been indicated by combining a CDK4/6 inhibitor.

We encountered a unique case involving gastric and colonic metastases originating from breast cancer. Here, we present this case to highlight the cytoreductive effect of CDK4/6 inhibitors for gastric and colonic metastatic lesions observed during upper and lower endoscopy. The patient provided informed consent for the presentation of anonymized clinical information.

## Case presentation

A 50-year-old female experienced intermittent pain in the epigastrium and entire lower abdomen over the course of several weeks, without diarrhea and hematochezia. In light of the persistent symptoms with no discernible improvement, she was referred to the Tokyo Bay Urayasu Ichikawa Medical Center. Fifteen years prior, she had undergone a left mastectomy while preserving the pectoral muscle and had received axillary lymph node dissection as part of her breast cancer treatment. The pathological results had revealed invasive lobular carcinoma, characterized by estrogen receptor (ER)-positive, progesterone receptor (PgR)-positive, human epidermal growth factor receptor 2 (HER2) 1+, with a clinical stage of pT2N1aM0, categorizing the cancer as stage ⅡB. Following surgery, the patient had received a 10-year course of hormone therapy with tamoxifen. She underwent routine breast ultrasound examination and mammography during and after the hormone therapy and had no recurrence of breast cancer, including in the contralateral breast.

**Figure 1:**
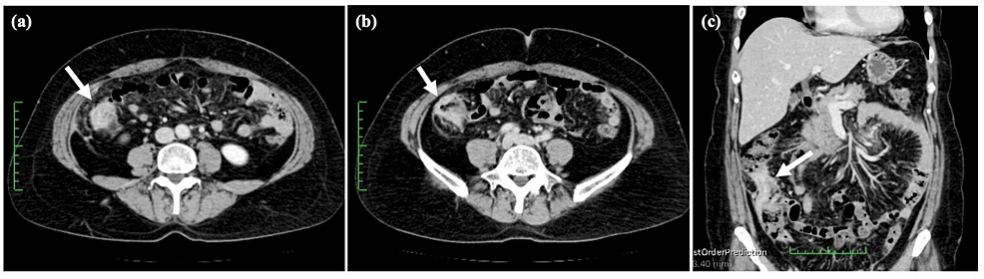
Abdominal CT images at diagnosis. Abdominal CT (a, b, c) shows the pronounced thickening of the bowel wall in the ascending colon (indicated by the arrow).

**Figure 2:**
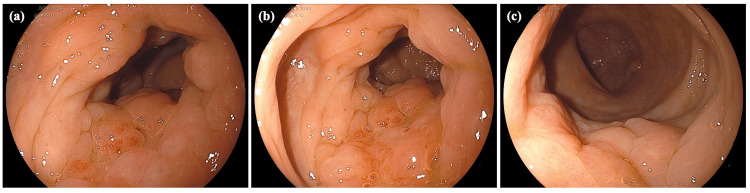
Colonoscopy image at diagnosis. Colonoscopy illustrating edematous folds and mild stenosis of the ascending (a, b) and sigmoid colon (c).

**Figure 3:**
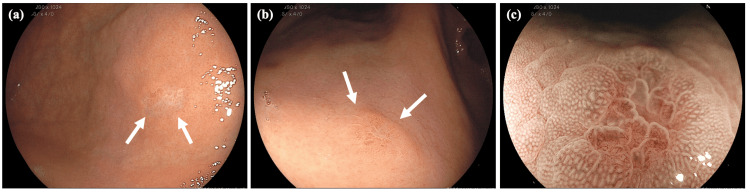
Upper endoscopy image at diagnosis. Upper endoscopy reveals discolored and depressed lesions in the anterior wall of the fornix (a) and the angulus (b) (indicated by the arrow). Magnifying blue laser imaging reveals an irregular surface pattern with irregular microvasculature in the depressed area of the angulus (c).

Presently, an abdominal computed tomography (CT) scan showed a thickening of the bowel wall in the ascending colon (Figure 1). The laboratory data yielded the following results: carcinoembryonic antigen (CEA) at 17.1 ng/mL (normal range = 0-5.0 ng/mL), cancer antigen 19-9 (CA 19-9) at 47.6 U/mL (normal range = 0-37 U/mL), and cancer antigen 15-3 (CA 15-3) at 68.5 U/mL (normal range = 0-30 U/mL). Colonoscopy findings indicated the presence of edematous folds and mild stenosis of the ascending and sigmoid colon (Figure 2). Biopsies were subsequently performed on the areas displaying mucosal edema and erosion of the ascending and sigmoid colon. Additionally, upper GI endoscopy revealed multiple discolored and depressed lesions in the stomach (Figure 3).

**Figure 4:**
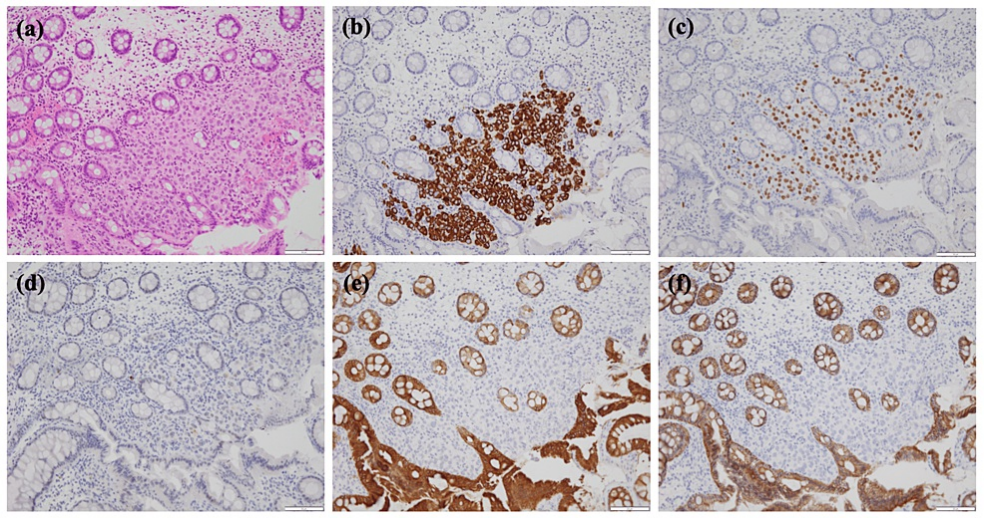
Histological examinations of the ascending colon specimen. Hematoxylin and eosin staining demonstrates the presence of poorly differentiated adenocarcinoma with signet ring cell within the mucosal tissue (a). Immunohistochemistry results reveal positive staining for cytokeratin 7 (b), estrogen receptor (c), and gross cystic disease fluid protein 15 (d), while showing negative staining for cytokeratin 20 (e) and E-cadherin (f).

Biopsies were similarly conducted on the depressed lesions, similar to the colonic lesions. The pathology results of the biopsy specimens from both the gastric and colonic lesions revealed poorly differentiated adenocarcinoma with distinctive signet ring cell features (Figure 4a). Immunohistochemical (IHC) staining of the colonic lesion further confirmed that tumor cells were positive for cytokeratin 7 (CK7), GATA-binding protein 3, ER, and gross cystic disease fluid protein 15 (GCDFP-15) and negative for cytokeratin 20 (CK20) and E-cadherin (Figures 4b-4f). Furthermore, the IHC staining of the gastric lesion showed the same pattern as that of the colorectal lesion. These results were consistent with breast cancer, particularly indicative of lobular carcinoma. Additionally, the IHC staining result was ER-positive, PgR-positive, and HER2-negative (1+).

**Figure 5:**
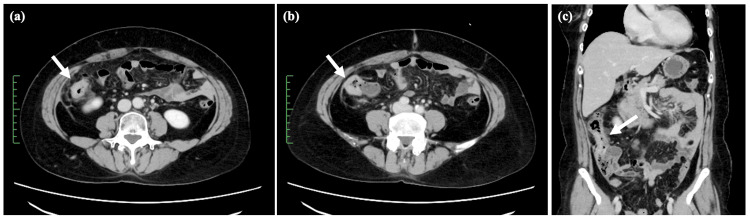
Abdominal CT images after anastrozole and palbociclib therapy. A metastatic lesion in the ascending colon (indicated by the arrow) shows no obvious tumor enlargement (a, b, c).

**Figure 6:**
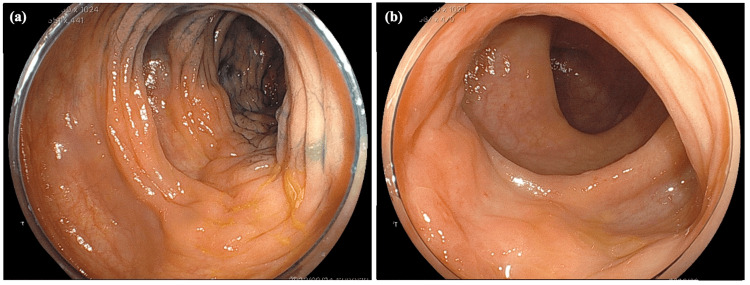
Colonoscopy image after anastrozole and palbociclib therapy. Colonoscopy shows a reduction of edematous folds and the disappearance of intestinal stenosis of the ascending (a) and sigmoid colon (b).

**Figure 7:**
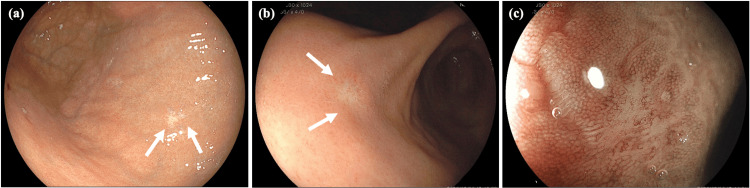
Upper endoscopy after anastrozole and palbociclib therapy. Upper endoscopy reveals a reduction and indistinctness of the metastatic lesion in angulus (a, b) (indicated by the arrow). Magnifying blue laser imaging shows a shrinkage of an irregular surface pattern with irregular microvasculature in the tumor lesion (c).

Thus, based on the above results, the patient was diagnosed with gastric and colonic metastases of breast cancer. Given that the patient was postmenopausal at the time of recurrence, she was prescribed a daily dose of 1 mg of anastrozole and 125 mg of palbociclib, administered for 21 days followed by a seven-day break in a 28-day cycle. Subsequently, the palbociclib dosage was reduced from 125 mg to 100 mg due to neutropenia. However, the patient was able to continue the treatment without any other adverse events. The initial CT scan performed in month two showed no obvious tumor enlargement of the ascending metastases, as defined by the Response Evaluation Criteria in Solid Tumors version 1.1 (Figure 5). Furthermore, three months into the treatment, GI endoscopy and colonoscopy were performed. Notably, colonoscopy showed a notable reduction in the edematous folds and the disappearance of intestinal stenosis (Figure 6). Similarly, GI endoscopy showed a reduction in the size of the multiple depressed lesions (Figure 7). For a duration exceeding one year up to the present date, the patient continued to receive treatment with anastrozole and palbociclib without any evidence of tumor progression, including in the stomach, colon, and bilateral breasts.

## Discussion

We present the rare case of a patient with gastric and colonic metastases originating from breast cancer. The patient received a combination treatment of ET and CDK 4/6 inhibitors. Notably, this combination therapy has demonstrated a superior antitumor effect compared to ET alone [5]. However, to date, there have been no reported instances of employing CDK4/6 inhibitors in the treatment of GI metastases from breast cancer. This case report not only challenges our current understanding but also holds the promise of unveiling novel treatment avenues. This report potentially marks the first case where we have been able to assess the morphological changes in GI metastasis induced by CDK4/6 inhibitors.

It is important to recognize that 10% to 40% of patients with breast cancer experience distant metastases within 5 to 20 years after breast cancer surgery [6]. The most affected sites include the liver, bones, and lungs. Conversely, GI metastases are uncommon, and previous studies have reported that the occurrence of gastric and colonic metastases is as low as 3% and 5%, respectively [7]. Moreover, simultaneous gastric and colonic metastasis is exceptionally rare, with only eight documented cases, including the case presented in our study [8]. Breast cancer, especially the luminal type, demonstrates a persistent tendency to recur, often spanning a prolonged period that sometimes exceeds 20 years. Therefore, it is imperative that we conduct a comprehensive inquiry into the patient’s medical history. Incidentally, lobular breast cancer is the predominant histological type found in GI metastases [9]. The reason for the higher rate of GI metastases in lobular breast cancer compared to ductal carcinoma has not been fully elucidated. However, there are assumptions that this is associated with the loss of E-cadherins and the GI tract microenvironment [9]. E-cadherins are cell-to-cell adhesion molecules that play a crucial role in maintaining cellular differentiation and preventing invasion. In lobular breast cancer, there is typically a loss of E-cadherin expression. Consequently, this subtype of breast cancer tends to exhibit increased dedifferentiation and invasiveness. Furthermore, the microenvironment of the GI tract is believed to be conducive to the growth of secondary tumor cells, as it not only provides the essential building blocks for tumor proliferation but also possesses a microanatomy that favors the entrapment of tumor cells [10].

The clinical manifestations of GI metastasis are characterized by non-specific symptoms such as intermittent abdominal pain, abdominal bloating, nausea, and diarrhea [11], while the endoscopic features of GI metastasis vary significantly and may include ulcers, mucosal edema, poor bowel expansion, and mild constrictive features [12]. Gastric metastases from breast cancer are sometimes solitary and present similar endoscopic findings to that of primary gastric cancer; therefore, differentiation between primary gastric cancer and metastatic lesions holds paramount importance [12]. In cases of metastatic breast cancer lesions, the malignant tissue tends to be confined to the submucosa and mucosal layers, increasing the possibility that tumors may evade detection through superficial biopsies alone. Consequently, a more comprehensive approach involving deeper biopsies is imperative to secure an accurate diagnosis [13].

Histological examination plays a pivotal role in diagnosing GI metastasis originating from breast cancer. However, this diagnosis can be complicated by the presence of signet ring cells, making it challenging to differentiate between primary gastric cancer and metastatic breast cancer [14]. Immunohistochemistry is a valuable tool for achieving a definitive diagnosis that differentiates between primary and metastatic lesions. This differentiation can be accomplished by evaluating the expression patterns of CK7, CK20, CDX2, ER, and GCDFP-15, as well as the loss of E-cadherin [15]. Breast and gastric cancers are both positive for CK7 and negative for CK20, but only breast cancer is positive for ER and GCDFP-15. It is important to note that even in primary colorectal cancer, ER is positive in approximately 30% of cases [16]. Therefore, establishing a definitive diagnosis necessitates a comprehensive evaluation based on multiple rounds of immunostaining.

The primary therapeutic options for unresectable or recurrent hormone receptor-positive, HER2-negative breast cancer are either ET or chemotherapy. When confronted with life-threatening metastases requiring an immediate antitumor effect, chemotherapy takes precedence as the primary treatment choice. On the other hand, due to its lower toxicity profile, ET is often the preferred initial treatment for advanced breast cancer [17]. In recent developments, first-line treatment recommendations for patients with postmenopausal breast cancer advocate combining aromatase inhibitors with CDK4/6 inhibitors, that is palbociclib and abemaciclib. Cyclin-dependent kinases (CDKs), such as CDK4 and CDK6, are enzymes that impair the regulation of cell proliferation in cancer cells, leading to uncontrollable proliferation [18]. CDK4/6 inhibitors exhibit antitumor effects by inhibiting these CKDs. Findings from randomized phase 3 trials, such as PALOMA-2, have revealed that patients with advanced breast cancer who received palbociclib in conjunction with letrozole treatment experienced significantly improved progression-free survival rates compared to patients who received letrozole treatment alone [2]. In addition, meta-analyses evaluating the benefits of adding CDK4/6 inhibitors to ET have demonstrated significant prolonged overall survival [19]. Similarly, this study reported a noteworthy finding: the combination of CDK4/6 inhibitors and ET yielded an overall response rate (ORR) approximately twice as high as that achieved with ET alone. The combination of CDK4/6 inhibitors and ET has demonstrated an ORR comparable to that of chemotherapy alone, making this combined approach effective in maintaining disease control, even in patients with breast cancer featuring visceral metastases [20]. However, it is crucial to note that the efficacy of CDK4/6 inhibitors in GI metastases of breast cancer remains uncertain. Consequently, the choice between CDK4/6 inhibitors and chemotherapy has not been firmly established in such cases.

## Conclusions

In this report, we presented a rare case involving breast cancer metastases to both the stomach and the colon. Through a comprehensive evaluation of endoscopic findings both before and after treatment with the combination of CDK4/6 inhibitors and an aromatase inhibitor, we highlighted noteworthy observations. This case underscores the potential of CDK4/6 inhibitors and ET to elicit a tumor-shrinking effect, even in GI metastases. To our knowledge, this is the first case to report such an antitumor effect by CDK4/6 inhibitors. While chemotherapy has traditionally been considered the primary treatment for visceral metastases even in hormone receptor-positive breast cancer, the advent of CDK4/6 inhibitors suggests that hormone therapy might be a feasible alternative in such cases.
